# Benefits of Metformin Combined with Pemetrexed-Based Platinum Doublets as a First-Line Therapy for Advanced Lung Adenocarcinoma Patients with Diabetes

**DOI:** 10.3390/biom11081252

**Published:** 2021-08-21

**Authors:** Jiun-Long Wang, Yi-Ting Tsai, Ching-Heng Lin, Abdulkadir Cidem, Theresa Staniczek, Gary Ro-Lin Chang, Chih-Ching Yen, Wei Chen, Kowit-Yu Chong, Chuan-Mu Chen

**Affiliations:** 1 Department of Life Sciences, and Ph.D. Program in Translational Medicine, College of Life Sciences, National Chung Hsing University, Taichung 402, Taiwan; wangjilong1217@gmail.com (J.-L.W.); cidema.kadir@gmail.com (A.C.); Theresa.Staniczek@medma.uni-heidelberg.de (T.S.); gary590422@yahoo.com.tw (G.R.-L.C.); 2 Division of Chest Medicine, Department of Internal Medicine, Taichung Veterans General Hospital, Taichung 407, Taiwan; 3 Division of Endocrinology and Metabolism, Department of Internal Medicine, Taichung Veterans General Hospital, Taichung 407, Taiwan; tinayttsai@gmail.com; 4 Department of Medical Research, Taichung Veterans General Hospital, Taichung 407, Taiwan; joelin99@gmail.com; 5 Department of Health Care Management, National Taipei University of Nursing and Health Sciences, Taipei 112, Taiwan; 6 Department of Public Health, Fu Jen Catholic University, Taipei 242, Taiwan; 7 Department of Dermatology, Venereology and Allergology, University Medical Center and Medical Faculty Mannheim, and Center of Excellence in Dermatology, Heidelberg University, 69117 Mannheim, Germany; 8 Department of Internal Medicine, China Medical University Hospital, and College of Health Care, China Medical University, Taichung 404, Taiwan; d5210@mail.cmuh.org.tw; 9 Division of Pulmonary and Critical Care Medicine, Chia-Yi Christian Hospital, Chiayi 600, Taiwan; peteralfa2004@gmail.com; 10 Department of Medical Biotechnology and Laboratory Science and Graduate Institute of Biomedical Sciences, Division of Biotechnology, College of Medicine, Chang Gung University, Taoyuan 333, Taiwan; 11 Hyperbaric Oxygen Medical Research Lab, Bone and Joint Research Center, and Department of Laboratory Medicine, Chang Gung Memorial Hospital, Linkou, Taoyuan 333, Taiwan; 12 The iEGG and Animal Biotechnology Center, and RongHsing Research Center for Translational Medicine, National Chung Hsing University, Taichung 402, Taiwan

**Keywords:** metformin, pemetrexed, lung adenocarcinoma, diabetes, first-line therapy

## Abstract

Lung cancer remains a challenge in daily practice. Chemotherapy is first considered for advanced lung adenocarcinoma bearing no active driver mutations. Maintaining drug efficacy and overcoming drug resistance are essential. This study aimed to explore the real-world use of anti-diabetic agent metformin in combination with pemetrexed-based platinum doublets in a first-line setting. We retrospectively collected data during 2004~2013 from TaiwaN′s National Health Insurance Research Database to access the survival benefit of metformin combined with pemetrexed-based platinum doublets as a first-line therapy for diabetic patients with advanced lung adenocarcinoma. Demographic data and information regarding platinum reagents, diabetes medications, and metformin doses were gathered, and overall survival status regarding metformin use was analyzed. Overall survival status based on the daily dose and the calculated cumulative defined daily dose (DDD) of metformin prescribed during the first 3 months after lung cancer was diagnosed was also assessed. A total of 495 patients were enrolled with a mean age of 67 years old, and the majority of the patients were male. After adjusting for age, sex, diabetes medication, and platinum reagents used, the adjusted hazard ratio (HR) for the metformin-user group was 0.61 (95% confidence interval (CI); 0.46~0.79; *p* < 0.001). The metformin-user group had a survival benefit (log-rank *p* < 0.001). We analyzed metformin dosing during the first 3 months after lung cancer diagnosis, and for a daily dose ≥ 1500 mg, the adjusted hazard ratio (aHR) was 0.42 (95% CI; 0.27~0.65; *p* < 0.001). Regarding the cumulative DDD of metformin, a DDD equal to or exceeding 21 resulted in aHR of 0.48 (95% CI; 0.34~0.69; *p* < 0.001). In this study, we found that the combination of metformin and pemetrexed-based platinum doublets provides a robust survival benefit as a first-line therapy for diabetic patients with advanced lung adenocarcinoma. It is worth conducting a large and randomized clinical trial to further investigate the antitumor effects of metformin on advanced lung adenocarcinoma when used as a first-ling therapy, including in non-diabetic patients.

## 1. Introduction

Lung cancer remains the leading cause of mortality in cancer patients worldwide, including Taiwan [1,2]. An increasing number of newly diagnosed lung adenocarcinoma has become predominant among non-small-cell lung cancer (NSCLC) patients [2]. For advanced lung adenocarcinoma without active driver mutations, such as epidermal growth factor receptor (EGFR) or echinoderm microtubule-associated protein-like 4-anaplastic lymphoma kinase (EML4-ALK) fusion mutations, instead of targeted therapy, chemotherapy with a platinum-based reagent is the main treatment option if there is no contraindication [3,4,5]. Immunotherapy is considered as a first-line therapy in patients with high expression of programmed death-1 (PD-1) or PD-ligand 1 (PD-L1) who are candidates for immune check point inhibitor (ICI) therapy [6,7,8,9]. Among chemotherapy reagent for advanced lung adenocarcinoma, pemetrexed (Alimta^®^) in combination with platinum derivatives as doublets is considered the first line treatment or is used alone for maintenance if tolerated by patients [10,11,12]. Pemetrexed has an impact on folate metabolism and serves as an antineoplastic agent. Clinically, the therapy is administered in 3-week cycles. The core issue of antitumor therapy is determining how to enhance drug efficacy while avoiding drug resistance, which is challenging in clinical practice.

Investigating and discovering new drugs requires a large budget and long-term development strategy. Approximately one in 5000 potential anticancer agents are approved by the US Food and Drug Administration (FDA) [13]. Currently available non-antitumor drugs could be repurposed for cancer management. Metformin was found to reduce the overall cancer incidence by 23% in the diabetic group [14] and exerted anticancer effects [15,16].

Metformin (N′,N′-dimethylbiguanide) is the most common oral antidiabetic drug (OAD) and is classified as a biguanide. Metformin reduces the risk for disease complications, such as cardiovascular disease, nephropathy, retinopathy, and polyneuropathy by correcting persistent hyperglycemia [17]. Currently, more than 120 million diabetic patients worldwide take metformin as a first-line blood glucose management regimen [18]. Metformin is relatively safe, inexpensive, and is associated with a lower incidence of lactic acidosis than that occurring with other OADs. Metformin induces a hypoglycemic effect by inhibiting gluconeogenesis. In recent decades, an increasing number of published articles have revealed an antitumor effect of metformin when used alone or in combination with other drugs in retrospective and meta-analysis studies [19,20,21,22,23]. Subgroup analysis revealed that metformin provides overall survival (OS) benefits in Asian individuals, those receiving chemotherapy, and patients with NSCLC, with pooled analysis hazard ratios (HRs) of 0.55 (*p* < 0.001), 0.58 (*p* < 0.001), and 0.75 (*p* = 0.03), respectively.

In lung cancer patients, patients treated with metformin had a potential survival benefit in comparison with those not treated with metformin in recent observational and national-based population studies [24,25,26,27,28,29]. In a previous study, Henderson et al. found that metformin use is related to improved 5-year survival rates in both colon cancer and lung cancer patients [29]. In addition, by utilizing the Surveillance, Epidemiology, and End Results (SEER) database, Lin et al. showed a survival benefit of metformin among a population of 750 diabetic stage IV NSCLC patients, and the HR was 0.80 (95% confidence interval (CI); 0.71~0.89) [28]. Even when combined with tyrosine kinase inhibitors (TKIs), metformin is still associated with improved survival benefits when metformin users are compared with non-metformin users [30]. When metformin is combined with chemotherapeutics and antiangiogenic agents in chemotherapy-naive advanced or metastatic non-squamous NSCLC patients, progression-free survival (PFS) rates are improved [31].

However, the effect of metformin combined with pemetrexed-based platinum doublets as a first-line advanced lung adenocarcinoma regimen has rarely been analyzed to date. Should the regular dose of metformin be maintained? Should the duration of metformin treatment be extended as long as possible during a period of chemotherapy with pemetrexed-based platinum doublets? Due to the lack of an obvious association between the dose response and time-dependent effects of combination therapy with metformin and pemetrexed-based platinum doublets after lung cancer diagnosis, in this study, we aimed to elucidate the trend of patient survival status associated with metformin use during chemotherapy. Furthermore, we aimed to clarify the beneficial and potential synergistic effects of metformin combined with pemetrexed-based platinum doublets for diabetic patients with advanced lung adenocarcinoma who received first-line chemotherapy in a cohort study.

## 2. Materials and Methods

### 2.1. Study Design

We retrospectively collected data from TaiwaN′s National Health Insurance Research Database (NHIRD) provided by the National Health Insurance Administration, Ministry of Health and Welfare, and managed by the National Health Research Institute of Taiwan (registered numbers 101095 and 102148). Patients with newly diagnosed lung cancer in Taiwan were selected for this study over a period of 10 years between 2004 and 2013. We focused on diabetic, chemotherapy-naïve advanced lung adenocarcinoma patients who received first-line chemotherapy with pemetrexed-based platinum doublets for lung cancer. The platinum derivative reagents included Cisplatin and Carboplatin. Patients who underwent surgery or targeted therapy (oral tyrosine kinase inhibitor (TKI)) prior to chemotherapy were excluded from the study. We divided diabetic patients depending on whether they used metformin. The use of medication for diabetes was defined as a treatment beginning at least 3 months before lung cancer diagnosis and persisting for at least 3 months after lung cancer diagnosis. We further calculated the daily dose (mg/day) and cumulative defined daily dose (DDD) of metformin during the first 3 months after lung cancer diagnosis. We wanted to explore the effect of metformin as adjuvant therapy in these patients and investigate possible synergistic effects of metformin combined with pemetrexed-based platinum doublets in a first-line chemotherapy setting. This study was approved by the Institutional Review Board (IRB) of the Taichung Veterans General Hospital (CE13151B-8). The informed consent was waived due to retrospective data collection.

### 2.2. Statistical Analysis

The baseline characteristics between metformin users and non-metformin users were compared using a chi-square test. To estimate the survival probability of metformin users and non-metformin users, we performed survival analyses using the Kaplan-Meier method, with significance determined by a log-rank test. Crude and adjusted hazard ratios (HRs) and 95% confidence intervals (CIs) for factors associated with survival were estimated using Cox proportional hazard regression models. All analyses were performed by SAS statistical software for Windows (version 9.3 for Windows; SAS Institute Inc., Cary, NC, USA), and the significance level was set at *p* < 0.05.

## 3. Results

### 3.1. Flow Chart of Patient Enrollment

**Figure 1:**
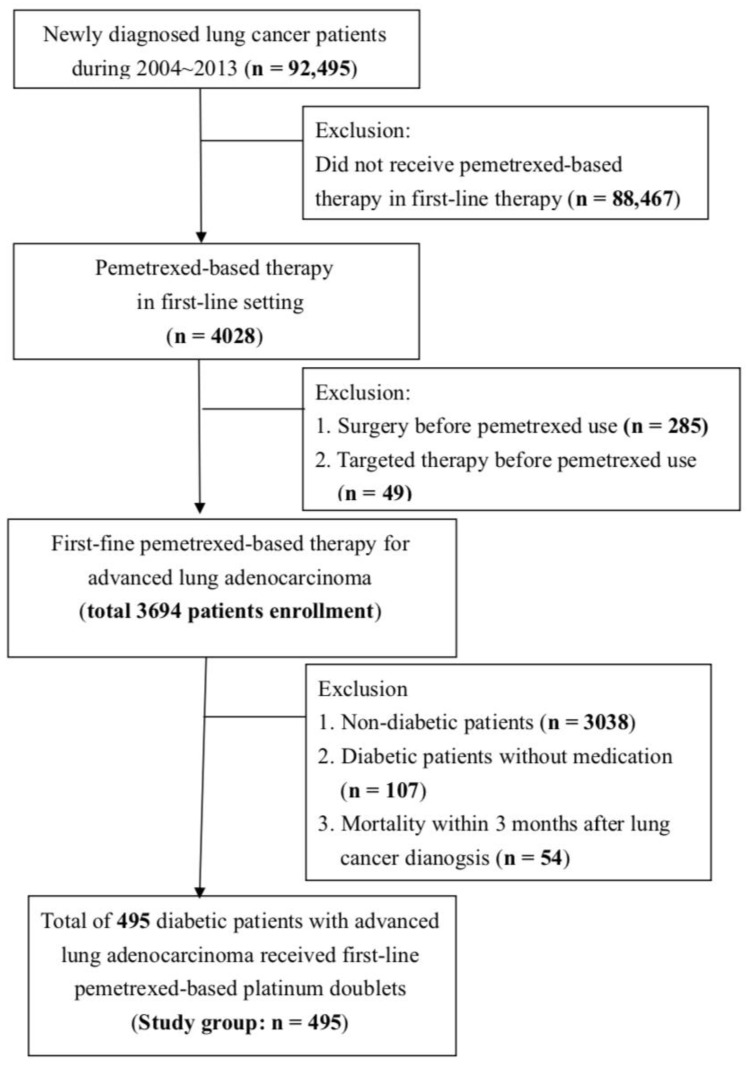
The enrollment flow chart of this study. Data were provided by the National Health Insurance Administration (NHIA) in Taiwan. Diabetic patients diagnosed with advanced lung adenocarcinoma receiving first-line chemotherapy with pemetrexed-based platinum doublets during 2004~2013 in Taiwan were enrolled in this study.

We used TaiwaN′s National Health Insurance Research Database (NHIRD) to analyze the effect of metformin combined with pemetrexed-based platinum doublets as a first-line treatment for diabetic advanced lung adenocarcinoma patients. Based on the flow chart (Figure 1), lung cancer patients newly diagnosed between 2004 and 2013 were initially identified for the study. In total, 92,495 newly diagnosed lung cancer patients were first investigated, including patients treated with pemetrexed that appeared in first-line reagent. Furthermore, we focused on diabetic patients who survived at least 3 months after the lung cancer diagnosis who had received first-line chemotherapy with pemetrexed-based platinum doublets. Finally, a total of 495 diabetic patients newly diagnosed with advanced lung adenocarcinoma receiving first-line pemetrexed-based platinum doublets were enrolled in our study.

### 3.2. Demographic and Baseline Characteristics of Our Study Group

A total of 495 patients were included in our study, and the baseline characteristics are shown in Table 1. The mean age was 67 years, and the patients were predominantly male. The mean age of the metformin user group was slightly lower than that of the non-metformin user group. The metformin user group consisted of a larger proportion of patients taking other treatments, including acarbose, sulfonylurea (SU), and thiazolidinedione (TZD). The distribution was not different according to platinum reagents (cisplatin or carboplatin) used. The use of statins was also verified, and there was no significant difference in statin use between these two groups. The comorbidity classification in metformin users and non-metformin users did not show a significant difference.

### 3.3. Overall Survival Status According to Diabetes Medication

We classified 495 diabetic lung adenocarcinoma patients according to the diabetes medication used, such as metformin, insulin, or others (including acarbose, SU, and TZD). The use of medication for diabetes was defined as a treatment beginning at least 3 months before lung cancer diagnosis and persisting for at least 3 months after lung cancer diagnosis. We also provided two models (model 1 was adjusted for age and sex and model 2 was adjusted for age, sex, OAD use, and platinum reagents use) to determine the adjusted hazard ratio (aHR) of each group. The aHR of the metformin user group by model 2 analysis was 0.61 (95% CI; 0.46~0.79), which was better than that of the non-metformin user group (*p* < 0.001). The aHR of the insulin group was 0.75 by model 2 analysis; however, the *p* value was 0.241. When the OAD consisted of acarbose, SU, or TZD, the aHR by model 2 analysis was 0.55, and the *p* value was less than 0.001 (Table 2).

### 3.4. Overall Survival Status of the Metformin User Group and the Non-Metformin User Group

**Figure 2:**
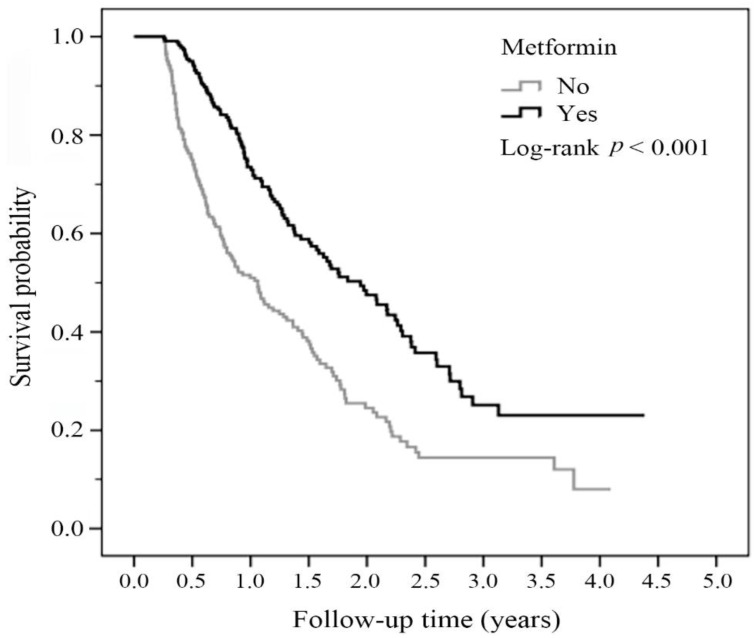
Overall survival status of the study groups classified by metformin and non-metformin treatment. The survival probability was analyzed by the Kaplan-Meier method during 4.0–4.5 years of follow-up. The *p* value for the log-rank test was *p* < 0.001.

We analyzed the overall survival status using the Kaplan-Meier method, and the significance was determined by a log-rank test. As shown in Figure 2, we found that the metformin user group had a better prognosis and improved survival outcome compared with the prognosis and survival rate of the non-metformin user group. The *p* value for the log-rank test was less than 0.001 (Figure 2).

### 3.5. Overall Survival Status Based on the Daily Dose of Metformin

**Figure 3:**
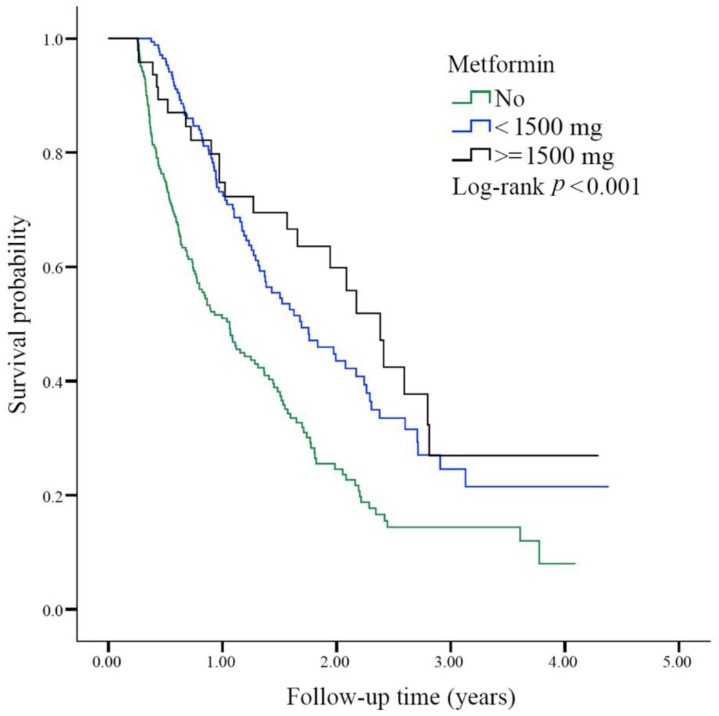
Overall survival status among the study groups according to the daily dosage of metformin prescribed during the first 3 months after lung cancer diagnosis. The survival probability was analyzed by the Kaplan-Meier method in the three different metformin prescription groups (0, <1500, and ≥1500 mg/day) of diabetic advanced lung adenocarcinoma patients. The log-rank test was *p* < 0.001.

We further analyzed the survival status of patients in each subgroup based on the prescribed daily dose of metformin during the first 3 months after lung cancer diagnosis. In the comparison between non-metformin users and metformin users, the aHR of the metformin user subgroup prescribed of a daily dose of less than 1500 mg was 0.52 (95% CI; 0.40~0.68; *p* < 0.001), and the aHR of the metformin user subgroup prescribed a daily dose of ≥1500 mg was 0.42 (95% CI; 0.27~0.65; *p* < 0.001). We determined survival status for each group of patients (no metformin vs. <1500 mg metformin vs. ≥1500 mg metformin). The *p* value of the log-rank test was less than 0.001 (Figure 3).

### 3.6. Overall Survival Status Based on the Cumulative Defined Daily Dose of Metformin

**Figure 4:**
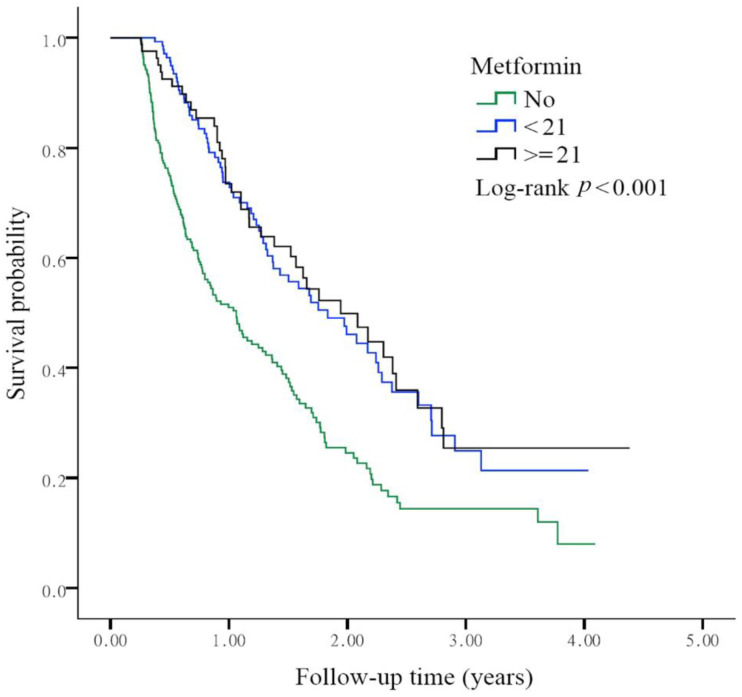
Overall survival status based on the cumulative defined daily dose (DDD) of metformin prescribed during the first 3 months after lung cancer diagnosis. The survival probability was analyzed by the Kaplan-Meier method in the three different groups (non-metformin user, the aHR for DDD < 21 and DDD ≥ 21). The log-rank test was *p* < 0.001.

We determined the survival status of each group based on the cumulative defined daily dose (DDD) of metformin during the first 3 months after lung cancer diagnosis. Compared with the non-metformin user group, the aHR of the metformin user group with a DDD less than 21 was 0.50 (95% CI; 0.38~0.67; *p* < 0.001). When the DDD reached or exceeded 21, the aHR was 0.48 (95% CI; 0.34~0.69; *p* < 0.001). The *p* value of the log-rank test was less than 0.001, and a survival benefit was observed in the metformin user group after adjusting for age and sex (Figure 4).

## 4. Discussion

In this study, we found that diabetic patients with advanced lung adenocarcinoma who received first-line therapy with pemetrexed-based platinum doublets and metformin had a better survival benefit than those who did not take metformin (Figure 2). To predict the protective role of metformin, we calculated the daily dose and cumulative DDD of metformin during the first 3 months after lung cancer diagnosis. Metformin was associated with improved survival outcomes in patients receiving a metformin dose of at least 1500 mg (Figure 3) and those with a DDD of 21 or higher (Figure 4).

Recent population-based studies demonstrated the survival advantage and benefit of metformin alone in lung cancer patients. In a previous study by the Brancher et al. [25] research group, the Norwegian Prescription Database was used to evaluate the OS and lung cancer-specific survival (LCSS) rates of patients who received metformin over a 10-year follow-up period (2005~2014). They found that metformin use after lung cancer diagnosis was associated with better LSCC rates, with a HR of 0.83 (95% CI; 0.73~0.95) in all patients, including adenocarcinoma and squamous cell carcinoma (SCC) patients [25]. The results of another Korean population-based nationally representative study (2004~2013) conducted by Kang et al. [27] showed that metformin use had a positive association with lower lung cancer incidences (*p* value = 0.008). In a study of TaiwaN′s National Health Insurance Research Database (NHIRD), Tseng et al. [20] reported that patients who had used metformin had a lower lung cancer incidence than those who had never used metformin after sample matching (211.71 vs. 292.65 per 100,000 person-years). The hazard ratio of the group who had used metformin was approximately 0.717 (95% of CI; 0.584 ~0.881).

When metformin was prescribed as adjuvant therapy with chemotherapeutic agents, we initially found an antitumor effect by a meta-analysis method approach [22]. Both improved disease-free survival (DFS) and OS rates were observed when metformin was combined with chemotherapy in the Asian NSCLC population. Marrone, et al. [31] conducted a phase II study of chemotherapy (carboplatin/paclitaxel/bevacizumab) with or without metformin in chemo-naive patients with advanced or metastatic NSCLC and showed improved progression-free survival (PFS) times (9.6 months vs. 6.7 months, *p* = 0.024). Lee et al. conducted a phase II study of chemo-naive patients with wild-type stage IIIB/IV NSCLC who received carboplatin and gemcitabine alone in combination with metformin (1000 mg twice daily). Although no obvious survival benefit was observed for unselected NSCLC patients, a survival benefit was observed in squamous cell carcinoma (SCC) patients with high fluorodeoxyglucose (FDG) uptake [32]. The HR for the death rate was 0.42 (95% CI; 0.18~0.94; *p* = 0.035). Another pooled analysis of a phase II study performed by Parikh et al. [33] revealed that the composite median PFS time for all analyzed patients was 6 months (95% CI; 1.36~7.96) when metformin was added to platinum-based chemotherapy with or without an antiangiogenic agent. 

We found two related studies of metformin in combination with pemetrexed-based platinum doublets as a first-line therapy. First, Parikh et al. initiated a pilot phase II trial to evaluate patients response to metformin combined with first-line chemotherapeutic reagents (carboplatin and pemetrexed) in non-diabetic patients with advanced stage NSCLC [34]. Although a small number of patients were enrolled, the study emphasized the lower cost and toxicity of this combination treatment and good patient tolerance to metformin and chemotherapy. Second, the goal of the fasting-mimicking diet and Metformin (FAME) is to explore the role of metformin and the fasting-mimicking diet (FMD) together with platinum-pemetrexed chemotherapy for advanced liver kinase B1 (LKB1)-inactivated lung adenocarcinoma. This trial is still underway. The primary assumption of this study is that the combination of the two experimental treatments improves the median PFS time from 7.6 months to 12 months when metformin is added [35]. 

Regarding drug delivery, determining the appropriate dose titration and maximizing drug exposure is crucial. The DDD was convenient to analyze [36]. Taking metformin as an example, Kang et al. used the time-dependent variable method as a representation of drug exposure intensity [27]. They found that metformin users had a lower incidence of lung cancer (*p* for trend = 0.008) and a lower mortality rate (*p* for trend < 0.001) than non-metformin users. The cumulative DDD of 547.5 mg of metformin was beneficial. A pilot study conducted by Parikh et al. gradually titrated the metformin dose each week from 1000 mg daily to 2000 mg daily. They also incorporated the concept of dosage titration [34]. In this study, we found that daily administration of 1500 mg of metformin and a DDD equal to or exceeding 21 reduced mortality in the advanced lung adenocarcinoma group (Figure 3 and Figure 4). In a study of patients with inoperable NSCLC, Chuang et al. analyzed nearly 3600 patients treated with metformin. The median dose of metformin was 140.56 mg DDD [26]. In the subgroup analysis, for patients who received chemotherapy, the metformin user group benefitted more, and the aHR was 0.86 (95% CI; 0.78~0.94). This finding is compatible with our results in this study (Figure 2).

How does metformin exert antitumor effect as a monotherapy or in combination with chemotherapeutic reagents? In previous studies, we found that metformin could inhibit tumorigenesis by an adenosine monophosphate-activated protein kinase (AMPK)-dependent pathway via inhibition of the mammalian target of rapamycin (mTOR) pathway [37,38,39]. The mTOR pathway is responsible for cell proliferation and protein synthesis. The targeting of the mTOR pathway and the subsequent antiproliferative effects are coregulated through LKB1. In addition, metformin could induce apoptosis by the AMPK-dependent PKA (protein kinase A)/GSK-3β (glycogen synthase kinase-3beta) pathway in NSCLC [40]. In an in vitro study of a lung cancer cell line provided by Zhang et al. [41], the combination of metformin with pemetrexed showed a synergistic anti-tumorigenesis effect by altering the cell cycle and enhancing apoptosis. Other AMPK-independent pathways, such as protein phosphatase 2 (PP2A), interferon regulatory factor-1 (IRF-1), hepatocyte growth factor (HGF), and autophagy, have been investigated [42]. Further efforts could be devoted to investigating the above mechanisms to further understand the antitumor function of metformin combined with pemetrexed.

## 5. Conclusions

In this study, we revealed that diabetic patients with newly diagnosed advanced lung adenocarcinoma who receive first-line pemetrexed-based platinum doublets chemotherapy benefit from metformin treatment. Patients treated with at least 1500 mg metformin and those with DDD equal to or exceeding 21 during the first 3 months after lung cancer diagnosis had better survival benefits. Further large-scale and randomized studies of all advanced lung adenocarcinoma patients, including non-diabetic patients, are required and anticipated. Our results provide an opportunity to further delineate a mechanism of metformin in combination with pemetrexed-based platinum doublets as a first-line treatment for patients with advanced lung adenocarcinoma without active driver mutations.

## Figures and Tables

**Table 1:** Baseline characteristics of study subjects (n = 495).

Variables	Total		Non-Metformin User (*n* = 270)		Metformin User (*n* = 225)		*p* Value
	*n*	(%)	*n*	(%)	*n*	(%)	
Age, mean ± SD	67.4 ± 9.5		68.3 ± 9.6		66.4 ± 9.2		0.026
<60	105	(21.2)	53	(19.6)	52	(23.1)	0.079
60–70	187	(37.8)	94	(34.8)	93	(41.3)	
≥70	203	(41.0)	123	(45.6)	80	(35.6)	
Gender							0.112
Female	214	(43.2)	108	(40.0)	106	(47.1)	
Male	281	(56.8)	162	(60.0)	119	(52.9)	
Diabetes medication							
Insulin	39	(7.9)	26	(9.6)	13	(5.8)	0.113
Acarbose/SU ^#^/TZD *	221	(44.7)	59	(21.9)	162	(72.0)	<0.001
Platinum reagents							
Cisplatin	373	(75.4)	199	(73.7)	174	(77.3)	0.351
Carboplatin	114	(23.0)	63	(23.3)	51	(22.7)	0.861
Statin	82	(16.6)	38	(14.1)	44	(19.6)	0.102
Comorbidity							
CVA ^1^	95	(19.2)	57	(21.1)	38	(16.9)	0.235
CAD ^2^	186	(37.6)	107	(39.6)	79	(35.1)	0.301
Congestive heart failure	40	(8.1)	25	(9.3)	15	(6.7)	0.292
COPD ^3^	222	(44.9)	126	(46.7)	96	(42.7)	0.373
CRD ^4^	22	(4.4)	12	(4.4)	10	(4.4)	1.000

Abbreviations: ^#^ SU: Sulfonylurea; * TZD: Thiazolidinedione; ^1^ CVA: Cerebral vascular accident; ^2^ CAD: Coronary artery disease; ^3^ COPD: Chronic pulmonary obstructive disease; ^4^ CRD: Chronic renal disease and it means serum creatine more than 2 mg/dL.

**Table 2:** Overall survival for study subjects according to diabetic medication.

Variables	Study Subjects		Model 1			Model 2		
	*n*	(%)	aHR	(95%CI)	*p* Value	aHR	(95%CI)	*p* Value
**Metformin**								
No	270	(54.6)	1.00	(reference)		1.00	(reference)	
Yes	225	(45.5)	0.50	(0.39–0.64)	<0.001	0.61	(0.46–0.79)	<0.001
*Insulin*								
No	456	(92.1)	1.00	(reference)		1.00	(reference)	
Yes	39	(7.9)	0.93	(0.59–1.47)	0.760	0.75	(0.47–1.19)	0.214
Others *								
No	274	(55.4)	1.00	(reference)		1.00	(reference)	
Yes	221	(44.7)	0.46	(0.36–0.60)	<0.001	0.55	(0.42–0.71)	<0.001

aHR: adjusted hazard ratio; Model 1: adjusted for age and gender; Model 2: adjusted for age, gender, OAD (oral antidiabetic drug), and platinum reagents (Cisplatin and Carboplatin); Others * include acarbose, sulfonylurea (SU), or TZD.

## Data Availability

The data presented in this study are available on request from the corresponding author.
